# Zenker diverticulum: Experience in surgical treatment of large diverticula

**DOI:** 10.1097/MD.0000000000010557

**Published:** 2018-05-11

**Authors:** Renata Tabola, Andrzej Lewandowski, Roberto Cirocchi, Katarzyna Augoff, Urszula Kozminska, Bartłomiej Strzelec, Krzysztof Grabowski

**Affiliations:** a Department of Gastrointestinal and General Surgery, Medical University of Wroclaw, Wroclaw, Poland; b Department of General and Oncological Surgery, University of Perugia, Perugia, Italy; c Department of Radiology; d Faculty of Medicine, Medical University of Wroclaw, Wroclaw, Poland.

**Keywords:** diverticulectomy, esophageal diseases, esophageal surgery, Zenker diverticulum

## Abstract

The purpose of this retrospective study is to show that transcervical diverticulectomy (TD) in treatment of Zenker diverticulum (ZD) can still be a first choice procedure in selected patients and in experienced hands its safety might be compared to the minimally invasive endoscopic diverticulostomy.

The study cohort consisted of 44 patients (18 male, 26 female) operated for (ZD). All the patients underwent open diverticulectomy. The decision to choose open surgical repair depended on surgical risk, age of the patient, size of the diverticular septum (the distance between the top of the diverticulum and its bottom on barium study), and patient's preference.

Mean age of patients was 64.6 ± 11.9 years; range: 26 to 88 years. A total of 36.4% out of them finished 70 years. Postoperative mortality was nil. Two major complications (4.5%) requiring surgical intervention occurred: leak and hematoma.

Data were analyzed by *t* test for independent samples using Statistica 12.5 software. *P* value <0.05 was considered statistically significant.

Surgical treatment of patients with ZD should be individualized. Large Zenker diverticula with the septum longer than 6 cm should preferably be resected through an open approach because it is not possible to remove the septum completely during one-step endoscopic procedure and diverticulostomy creates a weak and large common cavity in the esophagus. Surgical repair is effective for all sizes of diverticula, but its most serious complications such as leakage or laryngeal nerve injury should be considered, especially in elderly patients with comorbidities. However, age alone should not be the main criterion if choosing the treatment option.

## 1 Introduction

Zenker diverticulum (ZD) is a pulsion diverticulum that occurs in a natural weakness: the triangular shaped area of the posterior wall of the hypopharynx, which is bordered by oblique muscle fibers of the inferior pharyngeal constrictor and the horizontal muscle fibers of the cricopharyngeal muscle and is called Killian triangle. The cricopharyngeus marks the beginning of the esophagus and is a part of the upper esophageal sphincter (UES).^[1–3]^ The wall of the pseudo-diverticulum consists of mucosa and submucosa that bulge as a result of increased intraluminal pressure caused by incoordination between these muscles, and as a consequence of incomplete relaxation of the cricopharyngeus and the UES in a swallowing reflex. Cricopharyngeal myotomy remains a key element of surgical treatment of Zenker diverticulum (ZD).^[2,4]^

Although there is a lack of randomized clinical trials, surgeons favor minimally invasive endoscopic techniques – stapling or laser diverticulostomy – because they are believed to cause fewer complications and produce similar outcomes to classic transcervical repair.^[5–8]^

The purpose of our retrospective study is to show that transcervical diverticulectomy (TD) can still be a first choice procedure in selected patients and in experienced hands its safety might be compared to the minimally invasive endoscopic diverticulostomy (ED).

## 2 Material and methods

### 2.1 Patients’ selection

The study cohort consisted of 44 patients (0.7 male/female) operated on in the Department of Gastrointestinal and General Surgery of the Medical University of Wroclaw between January 2007 and December 2016. None of the patients was previously treated for ZD. All the patients underwent open diverticulectomy. The decision to choose open surgical repair depended on surgical risk, age of a patient, size of the diverticular septum (the distance between the top of the diverticulum and its bottom on barium study), and preference of the patient, after detailed explanation of the advantages and disadvantages of the surgery. Demographic and clinical data of the patients are presented in Table 1. All the patients underwent a barium study and the size of the diverticulum was evaluated. Patients younger than 70 years old (53.7% of our patients), excluding 1 patient who had the biggest diverticulum with the septum size of 15 cm, also underwent gastroscopy to evaluate esophageal and gastric mucosa primarily for gastroesophageal reflux disease and to exclude cancer. All the patients on fool oral diet after the operation were asked if they would undergo the diverticulectomy again under the similar postoperative results. Also we always ask the question if the patient on normal diet experiences any problems in swallowing solid nor liquid food. The study protocol was approved by our university ethics committee (agreement number KB84/18).

**Table 1:**
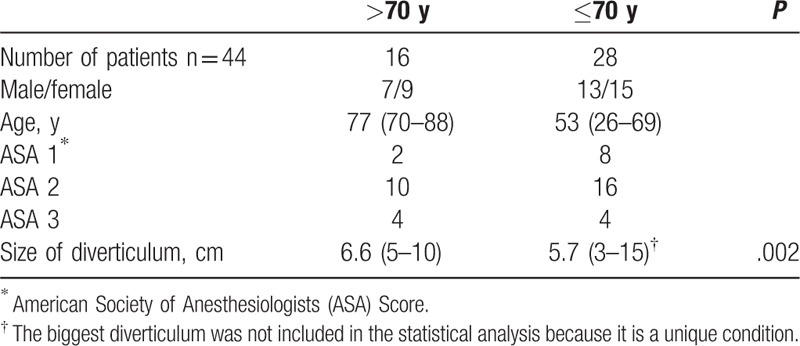
Demographic and clinical data of the patients’ population.

### 2.2 Open transcervical approach

The skin incision runs along the anterior border of the left sternocleidomastoid muscle. The platysmal flap is retracted medially and the sternocleidomastoid laterally. The omohyoid muscle is usually cut transversally and the middle thyroid vein is ligated to improve the approach to the pharyngo-esophageal border. The carotid sheath is carefully dissected laterally and the thyroid gland pulled medially, and finally the diverticulum that lies antero-medially to the prevertebral fascia is exposed. The recurrent laryngeal nerve (RLN) that runs in the tracheo-esophageal groove before it disappears in the cricothyroid muscle should be identified and preserved. The diverticulum is held in the clamp, pulled and dissected off its adhesions from the muscle fibers of the pharynx and the esophagus, until its bottom and neck are evident. We performed diverticulectomy and hand sutured the mucosal layer. Afterwards we approximated the muscular wall of the pharynx above the mucosa. Myotomy was performed after closing the esophageal lumen. Cricopharyngeal myotomy in our institution is an obligatory procedure and begins at the inferior border of the diverticular neck and extends onto the muscularis propria of the esophagus. The suction drain was placed parallel to the suture line and the platysma and the skin were approximated.

### 2.3 Postoperative care

After the operation patients were fed with a nasogastric tube located in the stomach during the surgery, or intravenously for 4 to 5 days until the soluble contrast was administered orally to rule out a leak from the suture line. An infected wound might also be indicative of a leak. If a leak was excluded a regular diet was introduced. The drain for bleeding control was removed after 24 hours following surgery. We prefer intravenous nutrition for older patients for 4 to 5 days because, in our opinion, they poorly tolerate a nasogastric tube.

### 2.4 Statistical analysis

The results are expressed as mean (±SD). Data were analyzed by *t* test for independent samples using Statistica 12.5 software. *P* value <.05 was considered statistically significant.

## 3 Results

**Figure 1:**
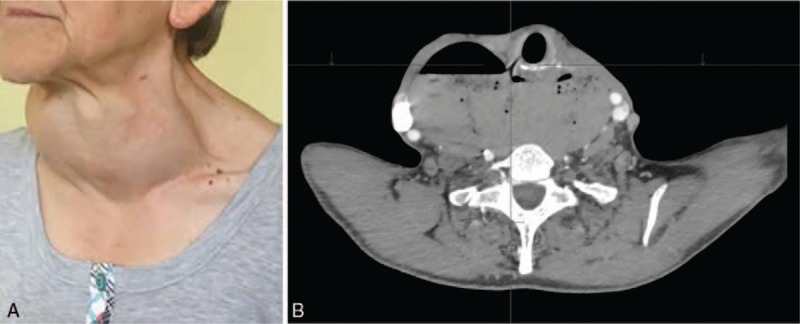
(A, B) Sixty three-years old women with the diverticulum with 15 cm long septum and esophgo-tracheal fistula. The diverticulum was permanently visible even she vomited and emptied it, and mimicked a large goiter. Computed tomography (CT) image of this large diverticulum with visible fistula between the esophagus and the trachea, which is abnormally translocated by the diverticulum permanently filled with air.

Mean age of patients was 64.6 ± 11.9 years (range: 26–88 years). Most patients with ZD are in their 7th or 8th decade of life and have multiple comorbidities that increase surgical risk. In our material 36.4% of patients were over 70 years old. However, the older patients (age range between 70 and 88 years, mean age: 76.7 years) tended to have large diverticula and were candidates for open repair in our opinion (*P* = .02). The septum size in the older patients’ group ranged from 5 to 10 cm (mean size: 6.6 ± 0.3 cm). The diverticulum caused dysphagia and regurgitation in all the patients (n = 44, 100%) and obviously difficulty in pharmacological therapy in many of them, because food and pills typically stuck in the diverticulum. The patients younger than 70 years old (mean age: 52.7 years; range: 26–69 years) presented with diverticula whose septa ranged between 3 and 15 cm; mean size of the septum was 6 ± 0.3 cm. A female patient with the biggest diverticulum, whose septum measured 15 cm, had a diverticulo-tracheal fistula. The fistula was confirmed on CT scans. We suspected tracheal fistula because the diverticulum was permanently filled with air (Fig. 1A, B). Thirty percent of younger patients also complained of halitosis. Endoscopy revealed chronic inflammation of the mucosa of the pyloric area in the majority of them and reflux esophagitis in 3 patients. The 2 youngest patients (26 and 42 years old) underwent esophageal manometry to exclude motility disorders.

Mean operation time was 80 ± 19 minutes (range 50–135 minutes).

Sixty-four percent of the patients underwent total parenteral nutrition for 4 to 5 days (mean 5 days, range 4–10 days) through a central venous catheter into the external carotid vein or ulnar vein.

**Figure 2:**
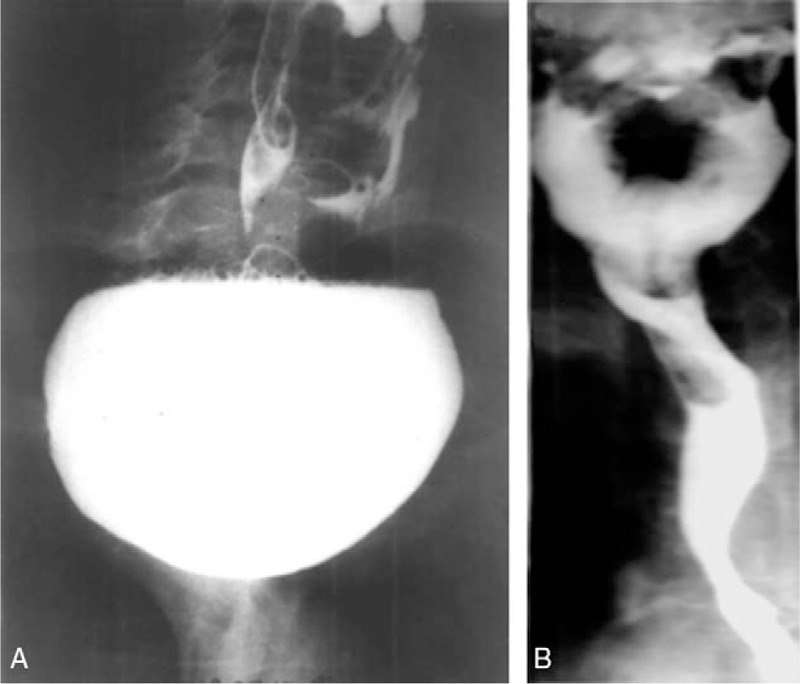
(A, B) Contrast radiogram of the esophagus presenting the diverticulum with the septum 12 cm long in the antero-posterior position. The same esophagus 6 months after diverticulectomy and myotomy. Antero-posterior position.

Postoperative mortality was nil. Two major complications (4.5%) occurred, 1 requiring surgical intervention: hematoma in a 69-year-old patient was evacuated on the first postoperative day; it did not have any further consequences. One patient developed an external pharyngeal fistula which resolved after 10 days with antibiotic treatment and total parenteral nutrition. One patient (2.3%) had a postoperative hematoma that resolved spontaneously. None had RLN injury, mediastinitis (including the patient who had leak after surgery), neck emphysema, or pneumonia postoperatively. Video contrast examination was obtained from all the patients and showed normal passage through the pharyngo-esophageal junction (Fig. 2A, B). Barium contrast in the patient, who developed leak, after its conservative treatment, did not show any stenosis (esophageal impression) or difficulty in passage through the pharyngoesophageal junction and the esophagus. The diverticulo-tracheal fistula in 1 patient was 2 mm wide and was approximated with 2 soluble sutures after dissection of the diverticulum. The course was also uneventful apart from transient left recurrent laryngeal nerve palsy (RLNP). Video contrast examination revealed no contrast penetration to the larynx.

All the patients were satisfied with the procedure including the one who developed leak and the one with the largest diverticulum and RLNP, and answered they would choose the operation again under the same postoperative results. None of our patients experienced difficulty in swallowing neither solid, nor liquid food at the time they left the word. Histological examination of the excised diverticulum confirmed chronic inflammation of the mucosa in all the cases.

## 4 Discussion

Current literature on treatment of ZD favors endoscopic over the external approach, but there are still indications for TD.^[9–14]^ Our patient population was not typical. The ratio of male to female was lower (0.7) and average diverticular size (6 cm) was bigger than in other study groups.^[1–3,8–10]^ This was a result of the selection of patients we made for the external approach. The patients operated on in our department were qualified to surgery considering their age, cardiovascular risk, and ability to tolerate general anesthesia. They also preferred the TD after detailed discussion of its disadvantages and benefits. Above all, our major indication for the open approach was diverticular size. In our population of patients, size of the diverticulum was statistically significantly bigger in the patients older than 70 years. The result may suggest that there are still candidates for surgery among older patients and the age should not be the only criterion if choosing the treatment option. Data from the literature on the size of the diverticular septum are sparse and patients operated trans-orally have relatively small diverticula between 3 and 5 cm with septotomy up to 3 to 4 cm.^[5,7–11,13–15]^ We wanted to show that there is still quite a large population of patients who are doubtful candidates for the ED and that alternative open diverticulectomy remains safe, especially in centers that specialize in esophageal surgery. Partial septotomy in diverticula bigger than 5 cm proposed by some surgeons even if results in successful myotomy still fails to completely remove the septum, which in future may require one or more endoscopic interventions.^[4,6,16]^

In our opinion and according to the literature, the septum size of 6 cm or bigger remains controversial for ED because diverticulostomy creates a long adynamic segment of the upper esophagus. Furthermore, complete removal of the septum is hazardous. A long septum requires a longer incision, and sutureless techniques in such cases encounter a higher bleeding rate, while stapling technique results in a higher leak incidence.^[11–13,16]^ Endoscopic results tend to decline over time, and we think that incomplete septotomy is a significant factor responsible for the failure rate.^[17–19]^

Visosky et al observed that out of 15% patients who primarily were endoscopicly managed, 63.7% of the revisions were performed via open approach.^[20]^ In the study by Chang et al open approach was performed in 53.9% patients, while all the recurrences were managed transcervically.^[21]^ Similarly Koch et al has to confirm, transcervical approach was performed 6 times more often than planned preoperatively, almost half of the recurrences after primary ED could be treated only by open approach and also underlined that in 1 patient after endoscopic intervention, 2 revisions were necessary but only transcervical approach finally allowed to remove residual sack that even after complete myotomy was a persistence source of food-trap mechanism. This food-trapping residual sac may be the cause of symptoms in patients with prolonged retention of contrast medium in the pharyngeal pouch and impaired passage into the esophagus, even after complete cricopharyngeal myotomy and septotomy.^[22]^

Incomplete cricopharyngeal myotomy might be visible on radiographs as circopharynngeal impression. Hypopharyngeal dysfunction during contrast swallowing may indirectly suggest incomplete myotomy or appear after RLNP. On contrast radiograms pharyngeal dysfunction shows as laryngeal or tracheobronchial penetration of contrast.^[23]^

Two of our patients showed cricopharyngeal impression on early postoperative contrast esophagogram that appeared as a slight indention in the wall extending approximately on 1.5 to 2 cm distance without any clinical symptoms of dysphagia or regurgitation. None of our patients showed any other radiographic symptoms of pharyngeal dysfunction during first contrast examination, especially in the aspect of delayed empting of the pharynx and pharyngeal penetration of the contrast. There was no persistent septum of the diverticulum on any of the contrast studies, however, according to the literature persistent septum characteristic rather for ED does not predict the treatment failure, especially in early postoperative period.^[23]^

Ozgursoy and Salassa reported that a large diverticulum alone as a mass may cause outlet obstruction and lead to high intrabolus pressure, even though the UES tonus remains normal on manometry.^[16]^ Such a theory leads to the hypothesis that the naturally weak wall of the hypopharynx may play an important role in genesis of ZD and leaving the weak adynamic segment of the esophagus creates a favorable environment for recurrence. Besides, creation of a large common cavity after diverticulostomy favors endoscopic techniques over open diverticulectomy in manometry examination, because in such a big cavity small pressures are difficult to identify, even though the remaining septum exists and causes some outlet obstruction.^[16,24]^ According to van Overbeek an anatomical predisposition to weakness in Killian dehiscence has a predominant role in the formation of ZD.^[25]^ The major complications after open diverticulectomy are leak and RLNP. We believe that one of the key elements influencing leak rate is incomplete myotomy. The 2nd reason for leaks might be too radical excision of the mucosa of the diverticulum and tension at the suture line, while a small excess of the mucosa can be easily invaginated by approximation of the muscular layer. The only patient with a leak was our youngest patient with a relatively high cricopharyngeal resting pressure and gastroesophageal reflux disease, and incomplete myotomy was a highly probable cause of it, but we did not observe any symptoms of incomplete myotomy on video contrast examination (cricopharyngeal impression or laryngeal contrast penetration) after complete healing of the esophageal wound. However, the 2nd possibility was wound infection caused by untreated dental caries. Unfortunately, he did not appear for follow-up examination. Our leak rate was similar to metadata from the literature (2.3% vs 3%).^[12]^ RLNP is also a complication more related to the open approach, and data from the literature indicate that its frequency may reach up to 3%.^[12]^ We are of the opinion that it might be minimized in experienced hands. Higher complication rates were observed in the 1990s and those data require updating.^[5]^ Similarly, the duration of the operation should be verified. In our material, the average duration of diverticulectomy was comparable to data from the literature.^[12,26,27]^ However, we did not use a stapler and performed only diverticulectomy, which is a longer procedure than diverticulopexy or invagination.^[9]^

The open approach and diverticulectomy result in longer recovery and a longer time to introduce oral feeding, but we believe there are contraindications to diverticulopexy in diverticula bigger than 6 cm. In our opinion, the time to introduce oral intake after excision of the diverticulum should not be shorter than 4 to 5 days, the period a wound needs to form granulation tissue. One might discuss the way of postsurgery nutrition. The majority of patients are fed enterally via a nasogastric tube. Many of our patients, probably due to age, poorly tolerated the tube or removed it accidentally, and in many cases (66%) we resorted to parenteral nutrition. The reported mean length of hospitalization after diverticulectomy is 8 to 15 days.^[12,14]^ In our institution, the mean hospital stay was within these limits.

We did not observe any death after TD. In the literature a mortality rate up to 9.5% is reported, but again, the data come from the 1980s and presently, with tremendous progress in medical care, this figure may not be accurate.^[5,14]^

Finally, considering our results possible selection bias such as the surgeon's experience and patients’ selection should be mentioned.

To sum up, we believe that surgical treatment of patients with ZD should be individualized. The patients with low cardiovascular risk and a large diverticulum may be offered TD and myotomy. Large Zenker diverticula with the septum longer than 6 cm should preferably be resected through an open approach because it is not possible to remove the septum completely during one-step endoscopic procedure and diverticulostomy creates a weak and large common cavity in the esophagus. Any further endoscopic procedure or consequent open surgical intervention might be much more complicated than primary surgery.

## 5 Conclusions

TD has a different but arguably comparable pattern of complications and advantages than ED. Surgical repair is effective for all sizes of diverticula, but its most serious complications such as leakage or laryngeal nerve injury should be considered, especially in elderly patients with comorbidities. However, the age of a patient should not be the only criterion when the decision is made as to the treatment method.

## Author contributions

**Conceptualization:** Renata Tabola, Roberto Cirocchi, Krzysztof Grabowski.

**Data curation:** Renata Tabola, Katarzyna Augoff, Bartłomiej Strzelec, Krzysztof Grabowski.

**Formal analysis:** Katarzyna Augoff.

**Investigation:** Renata Tabola, Andrzej Lewandowski, Urszula Kozminska.

**Methodology:** Renata Tabola.

**Software:** Bartłomiej Strzelec.

**Supervision:** Renata Tabola.

**Validation:** Andrzej Lewandowski, Roberto Cirocchi.

**Writing – original draft:** Renata Tabola.

**Writing – review & editing:** Renata Tabola.
